# Improvement of AZ91 Alloy Corrosion Properties by Duplex NI-P Coating Deposition

**DOI:** 10.3390/ma13061357

**Published:** 2020-03-17

**Authors:** Jaromír Wasserbauer, Martin Buchtík, Jakub Tkacz, Stanislava Fintová, Jozef Minda, Leoš Doskočil

**Affiliations:** 1 Faculty of Chemistry, Brno University of Technology, Purkyňova 464/118, 602 00 Brno, Czech Republic; xcbuchtik@fch.vut.cz (M.B.); tkacz@fch.vut.cz (J.T.); fintova@fch.vut.cz (S.F.); xcminda@fch.vut.cz (J.M.); doskocil@fch.vut.cz (L.D.); 2 Institute of Physic of Materials, Academy of Science Czech Republic, Žižkova 22, 616 62 Brno, Czech Republic

**Keywords:** Ni-P coating, AZ91 magnesium alloy, electroless deposition, corrosion, electrochemical test

## Abstract

The corrosion behavior of duplex Ni-P coatings deposited on AZ91 magnesium alloy was studied. The electroless deposition process of duplex Ni-P coating consisted in the preparation of low-phosphorus Ni-P coating (5.7 wt.% of P), which served as a bond coating and high-phosphorus Ni-P coating (11.5 wt.% of P) deposited on it. The duplex Ni-P coatings with the thickness of 25, 50, 75 and 100 µm were deposited on AZ91 magnesium alloy. The electrochemical corrosion behavior of coated AZ91 magnesium alloy was investigated by electrochemical impedance spectroscopy and potentiodynamic polarization method in 0.1 M NaCl. Obtained results showed a significant improvement in the corrosion resistance of coated specimens when compared to uncoated AZ91 magnesium alloy. From the results of the immersion tests in 3.5 wt.% NaCl, 10% solution of HCl and NaOH and 5% neutral salt spray, a noticeable increase in the corrosion resistance with the increasing thickness of the Ni-P coating was observed.

## 1. Introduction

Due to low weight, magnesium and its alloys show great potential in the field of automotive, aerospace, and electrochemical applications [1,2,3]. Magnesium alloys have low density, high modulus, good strength to weight ratio and physical properties [4]. However, their use is limited because of low corrosion resistance and poor wear properties. The electrochemical treatment of magnesium alloy surface is difficult due to their high chemical affinity to acid aqueous solutions [5]. On the other hand, performed surface treatment, which can improve the corrosion and wear resistance of magnesium alloys, is very important for many applications. The electroless conversion or thermally sprayed coatings, organic or ceramic coatings, varnishes are appropriate representatives to improve Mg alloys’ surface resistance against the corrosive environment and mechanical damaging in the industrial applications [6,7].

The application of electroless Ni-P coating is a very promising option to satisfy the demands of industry. The applications of electroless deposited nickel coating as a protective layer increase. It is characterized by high hardness, good wear, and high corrosion resistance, and the coating is uniform with no restriction on the shape of deposited substrate [8].

As the literature indicates [7,8,9], Ni-P coating can be divided into three groups based on the phosphorus content: low-phosphorus Ni-P coatings containing 1–5 wt.% of P, medium-phosphorus Ni-P coatings containing 6–9 wt.% of P and high-phosphorus Ni-P coatings containing 10–13 wt.% of P. The properties of each individual group are completely different. Some studies [10,11] show that low-phosphorus Ni-P coatings in the as-deposited state are crystalline or microcrystalline. Medium-phosphorus coatings are microcrystalline or form a mixture of amorphous and microcrystalline phases. High-phosphorus Ni-P coatings are completely amorphous. Low-phosphorus coatings are characterized by higher hardness and wear resistance than high phosphorus ones [9]. However, high-phosphorus Ni-P coatings are characterized by excellent corrosion resistance due to the absence of grain boundaries and other inhomogeneities, which can serve as active sites for the corrosion attack [7].

Lo et al. [12] studied the effect of phosphorus content on electrochemical behavior of coated materials in 3.5 wt.% NaCl. The authors reported that the corrosion potential, E_corr_, was shifted to more negative values, and the anodic current density increased in the case of low-phosphorus Ni-P coatings when compared to high-phosphorus Ni-P coatings. The polarization curves also showed that with the increase of phosphorus content in the deposited Ni-P coatings, significant decrease in anodic current in neutral and alkaline NaCl solutions was observed. The same results were reached in the case of electrochemical impedance spectroscopy in neutral NaCl solution. Low-phosphorus Ni-P coating (4.8% P) reached the polarization resistance Rp of 8442 Ω·cm^2^, and medium-phosphorus coating (8% P) reached the polarization resistance of 14,301 Ω·cm^2^. The high-phosphorus coatings formed 11% and 12.8% P the polarization resistances of 18,530 and 25,383 Ω·cm^2^ reached, respectively. The same trend was observed in alkaline NaCl solution. These results, correlated to the values given in the work of Gu et al. [13] where high-phosphorus Ni-P coatings (11.5% of P) reached the lower corrosion current density and higher corrosion potential when compared to low- (2.4% of P) and medium-phosphorus (8.8% of P) ones.

It is self-evident that the corrosion resistance is not only dependent on the phosphorus content but also on the thickness of the coating [14,15]. The corrosion tests such as the neutral salt spray (NSS) and immersion tests are an integral part of the quality control of corrosion protection of the coatings produced on the substrate. A corrosive medium such as salt, acid or alkali solution can be used. Zhang et al. [16] studied the corrosion resistance of the medium-phosphorous Ni-P coating (5.6% P) deposited on AZ91 magnesium alloy by the immersion test in 10% HCl solution and by polarization measurement in 3.5% NaCl. The results indicate that the immersion time in 10% HCl increased with increasing thickness of coating. The Ni-P coating with a thickness of ~7 µm failed after less than 10 min whereas the Ni-P coating with a thickness of ~28 µm failed after 45 min. Based on the results of potentiodynamic measurement in 3.5% NaCl, a significant shift to more electropositive potential –0.781 V and a corrosion current density decrease to 17.79 µA·cm^−2^ were observed.

Zhang et al. [17] studied the corrosion behavior of duplex Ni-P/Ni-B coating on AZ91 alloy in 5% NaCl NSS. As the authors indicate, no noticeable degradation of the coating was observed after 50 h of the experiment.

The aim of this paper is the study of the corrosion resistance of duplex Ni-P coatings deposited on AZ91 magnesium alloy. The first layer is a low-phosphorus deposit, and the second layer is a high-phosphorus one. The corrosion behavior of the coating was determined by means of potentiodynamic measurements, electrochemical impedance spectroscopy in 0.1 M NaCl solution, immersion test in acid, neutral, alkali environment and neutral salt spray test. The implementation of these experiments in various environments provides a primary comprehensive view on the corrosion behavior of duplex Ni-P coatings on AZ91magnesium alloy.

## 2. Experimental Material and Procedures

AZ91 magnesium alloy samples with the dimensions of approx. 20 mm × 20 mm × 7 mm were used as a substrate. The chemical composition of the substrate material determined using Glow-Discharge Optical Emission Spectroscopy (GDOES) is listed in Table 1.

The samples of AZ91 magnesium alloy were ground using 1200 SiC grit abrasive papers and then washed in distilled water and isopropyl alcohol and dried by hot air. To remove impurities from the samples surface, degreasing in an alkaline solution bath as a pre-treatment was applied (Table 2). After degreasing and cleaning the samples in distilled water and isopropyl alcohol, the surface activation in acid solution followed. The samples were again cleaned in distilled water and isopropyl alcohol and dried.

After the pre-treatment, the samples were immersed into the low-phosphorus electroless nickel plating bath. Based on the coating time, the final thicknesses of created duplex Ni-P coatings were gained: 25, 50, 75 and 100 µm [9]. In the case of Ni-P coatings with required final thickness of 25 and 50 µm, the deposition of low-phosphorus interlayer proceeded for 1.5 h. The thickness of low-phosphorus Ni-P interlayer was 14 µm. The deposition of low-phosphorus interlayer for 2 h was used in the case of Ni-P coatings with the final required thickness of 75 and 100 µm. The thickness of low-phosphorus Ni-P interlayer was in this case of 18 µm. The samples with deposited low-phosphorus interlayer were then immersed into the NiChem HP 1151 (Atotech) high-phosphorus electroless nickel bath for a defined time, as stated in Table 2.

The microhardness of deposited layers of duplex Ni-P coating was measured using LECO AMH55 microhardness tester (Saint Joseph, MO, USA). The microhardness was measured according to the Vickers method under applied load of 25 g for 10 s. The measurement was performed on the coated sample cross-cut mounted into epoxy resin prepared by standard metallographic methods (grinding and polishing). 

The average elemental content and elemental mapping of deposited duplex Ni-P coating were determined using the Zeiss EVO LS-10 scanning electron microscope (SEM) (Carl Zeiss Ltd., Cambridge, UK) equipped with an energy-dispersive X-ray spectroscopy (EDS) Oxford Instruments Xmax 80 mm^2^ detector (Oxford Instruments plc, Abingdon, UK) and AZtec software analysis (version 2.4).

The short-time potentiodynamic measurements were performed in 0.1 M NaCl solution at room temperature using the Bio-Logic VSP-300 potentiostat/galvanostat (BioLogic, Seyssinet-Pariset, France). Measured samples with analyzed area of 1 cm^2^ served as a working electrode. Saturated calomel electrode (SCE) was used as a reference electrode and Pt wire as a counter-electrode. 

Open circuit potential (OCP) variations with time were recorded up to 10 min of exposure. Afterward, Tafel plot was obtained by carrying out partial potentiodynamic polarization in the potential range from −100 mV to 200 mV from OCP at the potential scan rate of 1 mV/s.

Electrochemical impedance spectroscopy (EIS) measurements were performed in 0.1 M NaCl solution at room temperature for 5 min, 1, 2, 4, 8, 12, 24, 48, 72, 96 and 168 h within the frequency range from 100 kHz to 10 mHz. The perturbation amplitude was set up to 5 mV. 

The immersion corrosion tests were carried out on samples with deposited Ni-P coatings with various thicknesses: 25, 50, 75 and 100 µm. The samples were immersed in three solutions at room temperature, including 3.5 wt.% NaCl solution, 10 wt.% NaOH solution and 10 wt.% HCl solution. The exposure time was set based on the evolution of hydrogen. The experiment was stopped after the first hydrogen bubble had arisen from the surface of immersed sample.

The neutral salt spray testing was performed on coated samples as well. The samples were exposed to 5 wt.% NaCl solution at the temperature of 35 °C in LIEBISCH SKB 400 A-TR (LIEBISCH Laboratortechnik, Germany) corrosion chamber. The NSS testing was conducted according to the ASTM B117.6 standard. All experiments were performed on three samples for each coating thickness.

## 3. Results and Discussion

### 3.1. Pre-Treated AZ91 Magnesium Alloy Samples’ Surface Morphology

**Figure 1:**
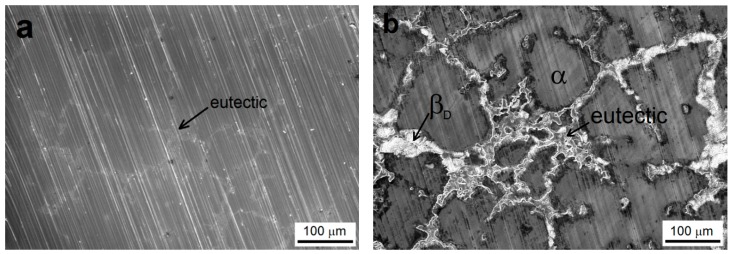
Morphology and structure of the AZ91 surface. (**a**) after grinding and degreasing, (**b**) after activation; SEM.

The surface of ground AZ91 magnesium alloy is shown in Figure 1a. The alkaline degreasing seems to have no significant influence on material surface roughness. Scratches after grinding are still obvious. On the other hand, the microstructure of the material was partially revealed; eutectic (α + β_D_ phase) areas can be identified. 

The etched surface of the AZ91 alloy after the immersion in the acid bath is shown in Figure 1b. The acid etching (surface activation) has revealed the microstructure of AZ91 alloy, and the presence of α solid solution grains, eutectic (α + β_D_ phase) and discontinuous precipitate β_D_ (Mg_17_Al_12_) was evident [18].

The surface pre-treatment of the AZ91 magnesium alloy has a great importance for the resulting quality of deposited Ni-P coatings and influences the corrosion properties of coated material [19,20,21]. Due to the galvanic coupling, preferred nucleation of Ni occurs on the areas of Mg_17_Al_12_ phase, and then, it spreads to the eutectics and next to α solid solution grains [22,23,24]. However, due to the heterogeneous phase composition of AZ91, the preferential nucleation of nickel on the areas of Mg_17_Al_12_ phase should lead to non-uniform Ni-P coating growth. As a result of non-uniform covering of the magnesium substrate surface, a lower quality of deposited Ni-P coating can occur.

### 3.2. Morphology and Chemical Composition of Deposited NI-P Coatings

**Figure 2:**
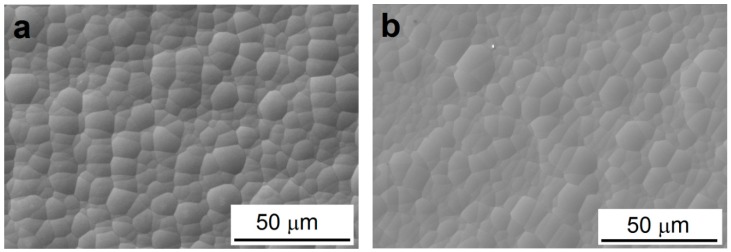
Surface morphology of Ni-P coating. (**a**) low-phosphorus interlayer, (**b**) high-phosphorus top coating; coating thickness 25 µm.

As can be seen in Figure 2, deposited Ni-P coatings exhibit spherical nodular structure without visible defects, no micropores in the case of both coatings, low-phosphorus Ni-P interlayer and high-phosphorus Ni-P topcoat can be observed. All coatings exhibited the same surface morphology and character. No coating structure coarsening was observed due to increasing coating thickness.

According to the literature [8,25] the porosity of Ni-P coatings decreases with increasing the thickness of coating. The electroless Ni-P coatings were shown to contain micropores in their volume when the coating time was lower than 80 min (thickness over 10 µm).

**Figure 3:**
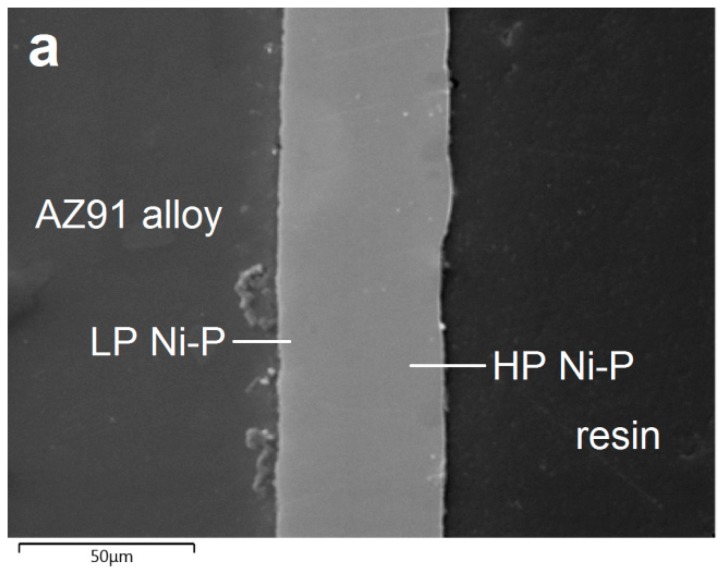

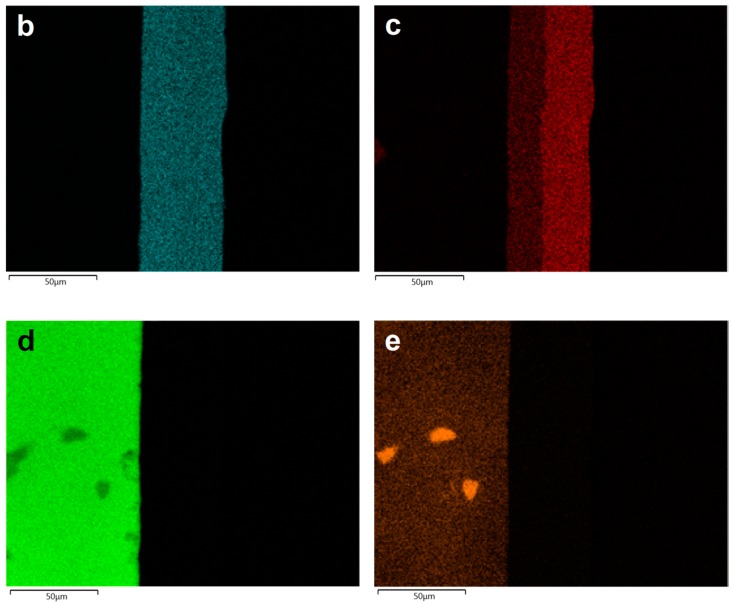
Micrograph (**a**) and EDS maps of deposited Ni-P coating on the AZ91 alloy: (**b**) Ni, (**c**) P, (**d**) Mg and (**e**) Al.

Figure 3 shows the cross-section micrograph of duplex Ni-P coating formed by a low-phosphorus interlayer with an average phosphorus content of 5.7 wt.% (~14 µm) and a high-phosphorus topcoat with an average phosphorus content of 11.5 wt.% (~36 µm). The deposited duplex Ni-P coating exhibits no oxide layer between AZ91 magnesium substrate and deposited Ni-P interlayer due to suitable pretreatment and deposition process. No visible defects or cracks were observed in the deposited Ni-P layer neither on the layer/substrate interface (Figure 3a). From the elemental EDS mapping (Figure 3), it was observed that each deposited Ni-P layer of duplex coating exhibits a homogeneous uniformity of chemical composition. The homogeneous composition of each layer of the duplex coating was provided by a sufficient amount of Ni^2+^ ions in the electroless nickel bath [7].

The areas of higher concentration of Al were observed on the Mg alloy surface. They can be ascribed to the presence of Mg_17_Al_12_ phase in the substrate microstructure (Figure 3e).

### 3.3. Microhardness of Deposited Coatings

The microhardness of deposited low-phosphorus Ni-P interlayer and high-phosphors Ni-P topcoat of duplex Ni-P coating, measured on the polished cross-section, was determined to be 620 ± 20 HV 0.025 and 580 ± 10 HV 0.025, respectively. The microhardness of cast AZ91 magnesium alloy was determined to be 70 ± 10 HV 0.025. It is evident that the low-phosphorus Ni-P interlayer has higher microhardness when compared to the high-phosphorus Ni-P coating. As mentioned in the literature [7,26], observed microhardness difference can be explained by the character of microstructure of the coatings. The coatings with higher phosphorus content are amorphous, as described in [7,27]. In the case of crystalline (low-phosphorus) Ni-P coatings, the ability to deform the crystalline lattice is substantially lower than that of the amorphous (high-phosphorus) Ni-P coatings. These differences in the material response to the deformation/loading result in higher hardness of coatings with crystalline structure when compared to the amorphous ones [28]. Agarwala and Agarwala [11] state that the hardness of deposited Ni-P coatings is strictly affected by their elemental composition. In their study, the low-phosphorus Ni-P coatings (2–3 wt.% of P) reached the hardness of 700 HV, and the high-phosphorus Ni-P coatings (10–12 wt.% of P) reached the hardness of 510 HV. In agreement with their observations, also in the work of Czagany and Baumli [29], the microhardness of the coatings decreased with increasing phosphorus content. As the authors stated, the highest microhardness (634 HV0.01) was reached for the coating containing 3.67 wt.% of P while the lowest microhardness (363 HV0.01) was reached for the coating with 13.48 wt.% of P.

### 3.4. Electrochemical Polarization Measurements

**Figure 4:**
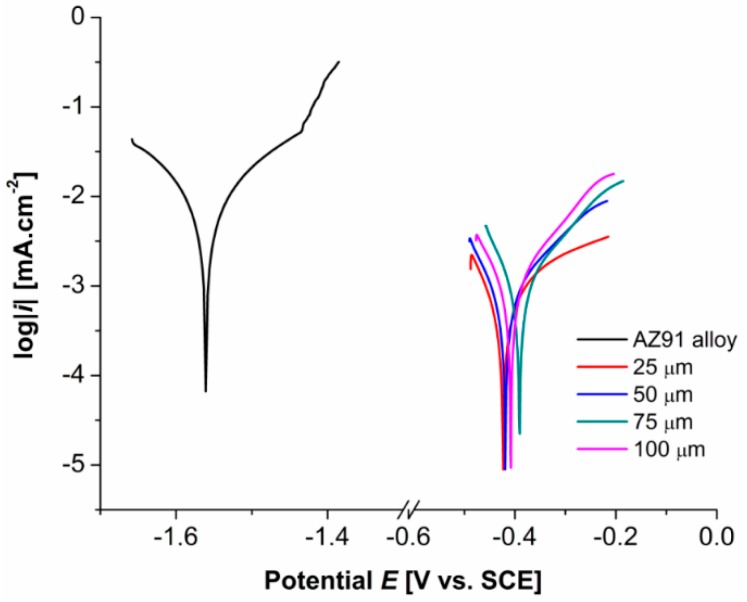
Typical potentiodynamic curves of AZ91 alloy and Ni-P coatings on AZ91 magnesium alloy in 0.1 M NaCl.

Figure 4 shows typical potentiodynamic curves of AZ91 magnesium alloy and deposited Ni-P coatings on AZ91 alloy in 0.1 M NaCl. Measured values of the corrosion potential, E_corr_, and the corrosion current density, i_corr_ were determined using the Tafel analysis, and their values are stated in Table 3. The samples of AZ91 alloy were ground (SiC no.1200 paper) directly prior to the measurement to remove the layer of oxides created on the surface of the sample due to the Mg reactivity.

For the AZ91 alloy, the hydrogen evolution (Equation (2)) is the main reaction on the cathodic branch of the polarization curve [3,23]. According to [30], AZ91 magnesium alloy dissolves in the corrosive aqueous solution forming Mg(OH)_2_ as described by Equations (1)–(3).

(1) $$\mathrm{Mg}\;\to \;{\mathrm{Mg}}^{2+}\;+\;{2e}^{-}$$

(2) $${2H}_{2}O\;+\;{2e}^{-}\;\to \;H_{2}\;+\;{2\mathrm{OH}}^{-}$$

(3) $${\mathrm{Mg}}^{2+}\;+\;{2\mathrm{OH}}^{-}\;\to \;{\mathrm{Mg}(\mathrm{OH})}_{2}$$

Created Mg(OH)_2_ compounds should form a protective layer against the following corrosion. This layer is insoluble in pure water but is not fully compacted and hence contains many micropores and microcracks. Moreover, in the presence of Cl^−^ ions in the solution, this layer is being constantly disrupted. As a result, MgCl_2_ soluble in the water solution (corrosive environment) is formed (Equation (4)) [3,21,31]. The formation of MgCl_2_ disrupts the compactness of protective Mg(OH)_2_ layer.

(4) $${\mathrm{Mg}(\mathrm{OH})}_{2}\;+\;{2\mathrm{Cl}}^{-}\;\to \;{\mathrm{MgCl}}_{2}\;+\;{2\mathrm{OH}}^{-}$$

This reaction results in the increase of pH value of corrosive solution due to the release of OH^−^ ions into the solution [19].

The pitting corrosion attack on AZ91 alloy samples can be observed on the anodic branch of the potentiodynamic curve, E_pit_ ≈ −1.4 V (Figure 4), where a relatively sharp increase in the corrosion current density occurred. 

It is evident that the E_corr_ of all specimens with deposited Ni-P coatings are significantly shifted to more electropositive values, and their polarization current density decreased by two orders of magnitude when compared to uncoated AZ91 alloy. The determined values characterizing the sample behavior form the thermodynamic and kinetic points of view are shown in Table 3. Prepared Ni-P coatings showed an increase in the corrosion resistance of the AZ91 magnesium alloy. 

From the thermodynamic point of view, based on the short-time potentiodynamic measurements, a significant improvement of corrosion properties of coated Mg alloy was determined. The values of the E_corr_ were determined for all coated materials to be almost the same. All coatings exhibit very similar protection against the corrosion. Moreover, no pitting attack was observed on tested coated specimens.

From the kinetic point of view, the corrosion rate of 25–75 µm thick Ni-P coatings is approximately the same. However, the lowest i_corr_ 0.358 µA·cm^−2^ was observed in the case of 25 µm thick duplex Ni-P coating, as shown in Table 3. A slight increase in i_corr_ and the decrease in E_corr_ was observed in the case of 100 µm thick Ni-P coating when compared to other coatings.

Further reduction of the corrosion current density and thus the corrosion rate of AZ91 alloy could be achieved, for example, by the co-deposition of ceramic particles in the Ni-P matrix [32]. If the ceramic particles are evenly distributed in the Ni-P matrix, the contact surface between the Ni-P matrix and the corrosive environment is reduced, which leads to the reduction of the corrosion rate. Hajiali Fina and Amadeh [33] observed that the Ni-P/nanoSiC composite coatings, deposited from the electroless nickel bath containing 15 g·l^−1^ of SiC nanoparticles, significantly improve the corrosion resistance of AZ91 magnesium alloy. The corrosion current density decreased from 0.13 mA·cm^−2^ for the uncoated alloy to 1.74·10^−6^ mA·cm^−2^ for the alloy with composite coating. The authors explained this fact by the theory that co-deposited SiC nanoparticles can change the corrosion path or even prevent its progress. The co-deposition of these nanoparticles in the Ni-P matrix can change the microstructure of the coating from columnar to coaxial. Moreover, present SiC nanoparticles can reduce the number of submicron defects in Ni-P coating and thus limit the penetration of the corrosive media to the coated substrate.

A significant reduction of E_corr_ and i_corr_ was observed also in the case of electroless Ni-P coatings with co-deposited carbon nanotubes (CNT) [34]. Plain Ni-P coating showed the corrosion potential of −0.411 V and the corrosion current density of 0.64 µA·cm^−2^. By the incorporation of CNT into the Ni-P matrix, the corrosion potential raised up to −0.292 V, and the corrosion current density decreased to 0.29 µA·cm^−2^.

### 3.5. Electrochemical Impedance Spectroscopy Characteristics

**Figure 5:**
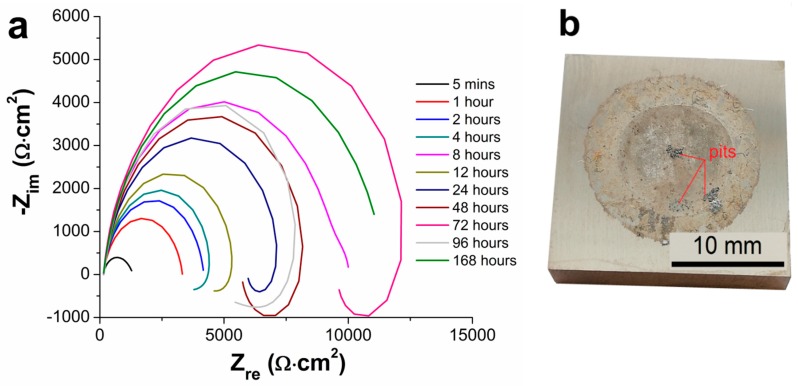
Nyquist plots of AZ91 magnesium alloy during measurement at different immersion times from 5 min until 168 h in 3.5 wt.% NaCl. (**a**) EIS of the uncoated AZ91 alloy and (**b**) sample of the uncoated AZ91 alloy after 168 h of exposition.

**Figure 6:**
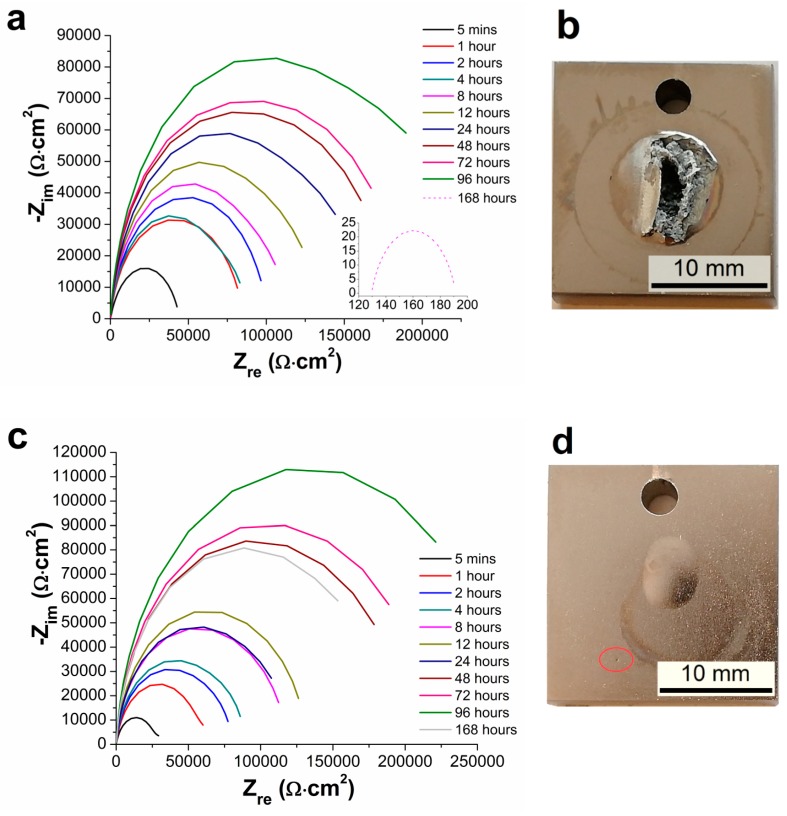

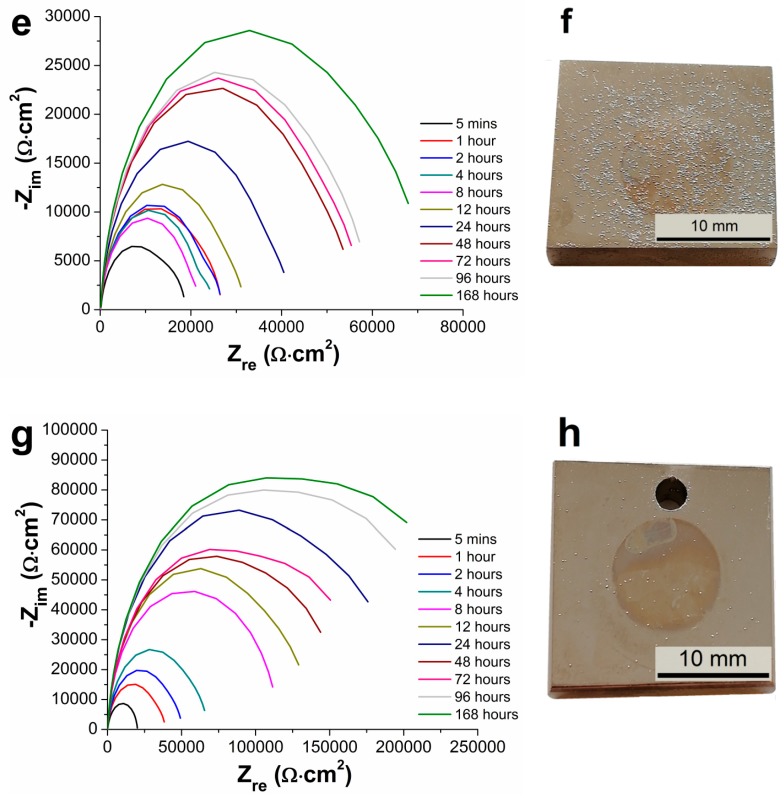
Nyquist plots of Ni-P coatings on AZ91 alloy during measurement at different immersion time from 5 min until 168 h in 3.5 wt.% NaCl. (**a**) EIS 25 µm, (**b**) sample 25 µm, (**c)** EIS 50 µm, (**d**) sample 50 µm, (**e**) EIS 75 µm, (**f**) sample 75 µm, (**g**) EIS 100 µm and (**h**) sample 100 µm.

Measured Nyquist plots obtained for the AZ91 magnesium alloy and Ni-P coatings on AZ91 alloy characterizing the long-time electrochemical corrosion behavior of the material in 0.1 M NaCl solution are shown in Figure 5 and Figure 6. Figure 5 shows the Nyquist plots of AZ91 magnesium alloy during 168 h of exposition. Figure 6 shows the Nyquist plots of AZ91 alloy with deposited duplex Ni-P coatings. In the case of 25 and 50 µm thick coatings, corrosion attack and local destruction of Ni-P coating were observed (Figure 6b,d), while in the case of 75 and 100 µm thick coatings no visible corrosion attack after 168 h (Figure 6f,h) was detected.

As seen in Nyquist plots, corresponding to the uncoated AZ91 alloy (Figure 5a), the increase in polarization resistance R_p_ was observed from the start of the experiment up to 8 h of the exposition. The decrease in R_p_ was observed at 8 h of exposition, where the value of R_p_ dropped from 9880 Ω·cm^2^ (8 h) to 3685 Ω·cm^2^ (12 h) (Table 4). This phenomenon is associated with the fact that the thin layer of Mg(OH)_2_ has been created on the specimen surface at the beginning of the exposition. The formed layer grew with the exposure time. However, this layer is chemically unstable in water solutions and in solutions containing Cl^−^ ions [3,30]. This passive layer broke down between 4 and 8 h of exposition, resulting in uncovering of the metallic substrate surface. The uncovered substrate immediately reacted with the corrosive media to form Mg(OH)_2_ and fill the defects in damaged layer [30]. This formation of the passive layer led to the following increase in R_p_ up to 72 h of exposition when the phenomenon was repeated. Figure 5b shows that a pitting occurred during the exposition in 0.1 M NaCl solution, which can be reflected in the inductive response of Nyquist plots (Figure 5a). The pitting is typical for the magnesium alloys [3,21,35]. The behavior is the consequence of different potential of Mg solid solution and of present intermetallic phase particles and eutectics, creating a galvanic couple [3,22].

The EIS plots for 25 and 50 µm thick coatings are shown in Figure 6a,c, correspondingly. The values of polarization resistance R_p_ increased with increased exposition time in NaCl solution up to 96 h. This increase in R_p_ is probably observed due to the reaction between Ni and corrosive environment, which led to the formation of passive film on the surface of the Ni-P coating. This film could provide a barrier against the corrosion attack on Ni-P coating and thus protect the coated AZ91 alloy. Nickel undergoes the passivation reaction in aqueous solutions forming several nanometers thick film of nickel oxide (NiO) or hydrated nickel oxide (NiO_x_H_y_) [36,37]. 

The corrosion attack on 25 µm thick duplex coating occurred in the interval from 96 to 168 h of exposition, where the destruction of Ni-P coating and the corrosion of AZ91 alloy occurred (Figure 6b). In the case of 25 µm thick coatings, the polarization resistance at 168th hr decreased from 233 570 Ω·cm^2^ (96 h) to 63 Ω·cm^2^ (168 h). This value is substantially lower when compared to the uncoated magnesium alloy. The drop can be explained by the galvanic coupling between Mg alloy and Ni-P coating [30]. Because the Ni-P coating was destroyed and the corrosive environment reacted with the substrate, the corrosion of the substrate was accelerated. The reaction was accompanied by the hydrogen evolution due to the reaction of Mg and corrosive solution.

In the case of 50 µm thick duplex Ni-P coating, formation of blisters (Figure 6d) during the measurement was observed. Since no damage of the coating was observed, no significant change in polarization resistance was observed during the measurement, and the value of R_p_ remained almost unchanged from 46 to 168 h of measurement.

Figure 6f shows 75 µm thick duplex Ni-P coating on the AZ91 alloy. It is obvious that the deposited coating was not completely homogenous before the NaCl exposition; it was rough, and visible protrusions could be observed. As presented in [2,38], these protrusions contain higher amount of structural defects [39]. Clearly, the corrosive solution can easily pass through these defects to the magnesium substrate, or it can be detained in pores of these protrusions. This phenomenon is then reflected in the value of polarization resistance R_p_ (Figure 6e). Due to the corrosion of the substrate as a consequence of the detained solution in the pores of Ni-P coating and structural defects, the passivation layer of NiO is disturbed or removed, resulting in the revelation of Ni-P coating.

In the case of 100 µm thick duplex Ni-P coating, no defects or blisters were observed (Figure 6h) on the sample surface after the measurement. Similar values of the polarization resistance in time determined for the samples are consistent with this observation.

**Figure 7:**
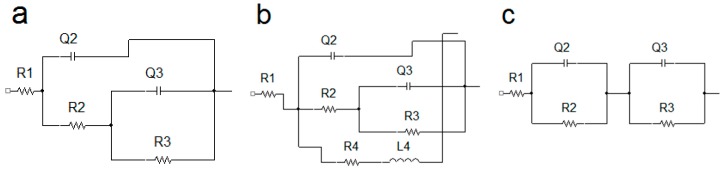
Equivalent electrical circuit models used for the EIS data analysis. (**a**) EC for the analysis of uncoated AZ91 alloy, (**b**) EC for the analysis of 25 µm thick Ni-P coating on the AZ91 alloy after 168 h and (**c)** EC for the analysis of all duplex Ni-P coatings.

To describe the electrochemical corrosion behavior of uncoated AZ91 alloy and AZ91 alloy with deposited Ni-P coatings in 0.1 M NaCl, the equivalent circuits (EC) simulating solution/sample interface given in Figure 7 were used. Figure 7a shows the EC model including two capacitive loops for high and low frequencies formed by capacities (Q) and their resistances (R); R_1_ (resistance of the solution), R_2_ (resistance of Mg(OH)_2_/NaCl solution interface) and R_3_ (resistance of metallic Mg and NaCl solution interface) [40]. This model describes the behavior of uncoated AZ91 alloy exhibited in 0.1 M NaCl solution for 5 min. The model corresponds to the formation of a thin layer of Mg(OH)_2_ on the metallic surface of AZ91 alloy [31]. However, formed Mg(OH)_2_ layer is not completely uniform due to its porous nature and is also damaged by cracks, and so the metallic surface of AZ91 alloy was in contact with the corrosive environment [3,21]. The resulting R_p_ value is the sum of R_2_ and R_3_ according to Equation (5).

(5) $$R_{P}=R_{2}+R_{3}$$

The EC model (Figure 7b) representing the electrochemical corrosion behavior of uncoated AZ91 alloy in 0.1 M NaCl solution after 5 min includes the elements such as R_1_, which is the solution resistance [41]. High-frequency loop characterizing damaged Mg(OH)_2_ layer is observed—elements Q_2_ and R_2_ (Figure 7b). Low-frequency loop describing the interface of the solution and revealed metallic substrate (defects in the protecting layer of corrosion products) is defined by elements Q_3_ and R_3_. Inductive loop characterized by elements L_4_ (inductance) and R_4_ is related to the description of negative difference effect (NDE) caused by the adsorption of ions (H^+^, Mg^+^ or Cl^−^) on the sample surface and evolution of gas H_2_ [3,42]. The resulting R_p_ value is calculated according to Equation (6).

(6) $$R_{P}=\frac{\left(R_{2}+R_{3}\right)\cdot R_{4}}{R_{2}+R_{3}+R_{4}}$$

The exposed sample of uncoated AZ91 alloy was heavily corroded, and the pitting was visible on the surface (Figure 5b) after 168 h in 0.1 M NaCl solution. During 168 h, the NDE effect and the adsorption of ions do not play such a big role as within 1–96 h; therefore, the EC model given in Figure 7a was used for the analysis of measured data again.

The behavior of the magnesium alloy in the corrosive environment was characterized by the creation of porous layer of Mg(OH)_2_. Only a low corrosion protection was provided by the formation of layer of corrosion products on the magnesium alloy surface. However, this porous layer did not protect the magnesium alloy against the corrosive medium sufficiently, and the corrosion process occurred. As a result, the lowest values of the polarization resistance were determined for untreated specimens when compared to coated samples, Table 4.

The behavior of all duplex Ni-P coatings deposited on the AZ91 magnesium alloy samples was analyzed by the EC model in Figure 7c, except the 25 µm coating after 168 h of the exposure to the corrosive environment [43]. In the case of the EC model shown in Figure 7c, R_1_ is the resistance of the solution, R_2_ is the resistance against the charge transfer between the electrolyte (NaCl solution) and upper high-phosphorus layer of the Ni-P coating, Q_2_ is the capacity of double-layer NaCl solution/ Ni-P coating, R_3_ is the resistance against the charge transfer between high-phosphorus and low-phosphorus layers of the Ni-P coating, and Q_3_ is the corresponding capacity. The resulting polarization resistance value is given by Equation (5) [31].

The EC model in Figure 7b was used for the analysis of the Nyquist plot for 25 µm thick Ni-P coating on the AZ91 alloy after 168 h in 0.1 M NaCl solution. The resulting polarization resistance value is given by the relation in Equation (6) [31].

The values of polarization resistance determined for the samples with 25, 50 and 100 µm thick coatings were comparable to each other, see Table 4, so it can be assumed that all layers offered the same protection against corrosion regardless of the coating thickness.

### 3.6. Corrosion Behavior during Immersion Tests

From the results of long-term immersion tests in 10% HCl and 3.5% NaCl solutions at room temperature, it can be seen that the corrosion resistance of coated samples increased with increasing thickness of deposited duplex Ni-P coatings (Table 5). For 100 μm thick duplex Ni-P coating no hydrogen bubble arising from the surface was observed in 10% HCl solution up to 24 h. For the sample with 100 μm thick coating immersed into 3.5% NaCl solution hydrogen bubble was observed for 792 h. 

The corrosion model describing the damage of Ni-P coating deposited on magnesium alloy is referred to in the article [2]. Hydrogen evolves during the deposition of Ni-P coating (Equations (7) and (8)) as a byproduct of the reaction, and produced hydrogen is adsorbed on the surface of growing coating. This phenomenon results in the formation of pores or channels in the coating through which the corrosive medium can potentially pass to the magnesium substrate [7,44,45].

(7) $${\mathrm{Ni}}^{2+}+{4H}_{2}{\mathrm{PO}}_{2}^{-}+H_{2}O\to {\mathrm{Ni}}^{0}+{3H}_{2}{\mathrm{PO}}_{3}^{-}+P^{0}+H_{2(\mathrm{ads})}+H\cdot +\;H^{+}$$

(8) $$2H\cdot \to H_{2}$$

When the coated magnesium-based substrate is immersed into the solution containing Cl^−^ ions, the solution can penetrate to the magnesium substrate surface through the defects in the coating. As a result, the corrosion cells are formed on the coated surface because the Ni-P coating acts as a cathode, and magnesium substrate acts as an anode and corrodes [37].

The improvement of corrosion properties of the substrate was achieved by the deposition of ternary Ni-P coatings. Zhang et al. [14] referred that deposited Ni-Sn-P coatings can increase the corrosion resistance of magnesium alloy in 10% HCl solution several times when compared to binary Ni-P coatings. For 35 µm thick Ni-Sn-P ternary coating deposited on the AZ91D magnesium alloy, no hydrogen bubbles arising from the surface after the immersion in 10% HCl solution for 608 min were observed. Meanwhile, the Ni-P coating with the same thickness protected the substrate only for 76 min (1.27 h). Zhang et al. [16] also stated that deposited 24 µm thick ternary Ni-W-P coating protected the substrate in 10% HCl solution for 187 min (3.12 h) without observing any hydrogen bubbles arising from the coated specimen surface. The Ni-P coating with the thickness of 28 µm protected the material only for 45 min (0.75 h). 

The corrosion resistance (exposition time) of all analyzed deposited duplex Ni-P coatings in 10% NaOH solution reached 1000 h (Table 5). Zeller a Salvati [37] reported that very low corrosion rates (≤ 2 μm/py) were measured for the Ni-P coatings in 50% NaOH solution at room temperature. The authors also concluded that the corrosion resistance of coated substrate in the NaOH solution is dependent upon the content of P in deposited Ni-P coatings. High-phosphorus Ni-P coatings have worse corrosion resistance in comparison with medium or low-phosphorus coatings. Low-phosphorus coatings have the corrosion resistance comparable to pure nickel. The explanation is that phosphorus present in the Ni-P coating may form soluble complexes of nickel (Ni_3_[PO_4_]_2_) on the coating surface in water. As a result, nickel forming the passive Ni(OH)_2_/NiO film is removed from coated substrate surface. The thickness of surface protective Ni(OH)_2_/NiO film is dependent on the P content [37,46].

Even though the top layer of experimental duplex coatings was formed by high-phosphorus coating and a low protection against the corrosion could be assumed, the exposition time of coated AZ91 specimens reached 1000 h. A good corrosion resistance could be reached due to quite high thickness of the layer and due to the formation of nickel oxide (NiO) or hydrated nickel oxide (NiO_x_H_y_) on the surface, protecting the material against the corrosion [37].

### 3.7. Neutral Salt Spray Exposure

According to the immersion tests, the exposition time in NSS increased with the increasing thickness of the duplex Ni-P coatings as well as for 10% HCl and 3.5% NaOH solutions (Table 5). Higher coating thickness increased the resistance of alloy against the corrosion. The individual for each coating are listed in Table 5.

**Figure 8:**
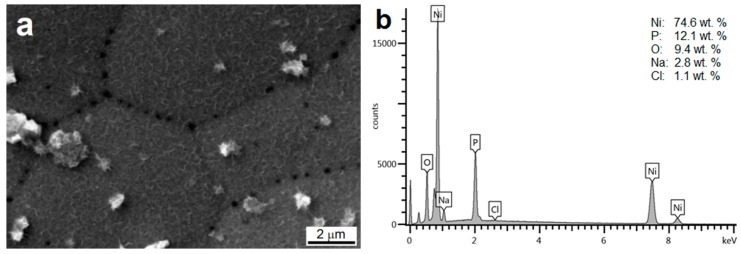
Example of the surface morphology of duplex Ni-P coating (100 µm) after the exposition in NSS for 430 h (**a**) and the surface EDS analysis (**b**).

The presence of pits on the Ni-P coating nodules boundaries is documented in Figure 8a. This phenomenon was observed for all coatings and was caused by the corrosion attack on the sample in the salt spray. The EDS analysis (Figure 8b) showed higher oxygen content (9.4 wt.%), which can suggest the presence of porous passive Ni(OH)_2_/NiO thin layer [37].

The mechanism of the corrosion attack on the Ni-P coated AZ91 alloy was the same as for the immersion tests. The micropores present in the Ni-P coating allow the transport of the corrosion media into the magnesium alloy surface. As a consequence, the magnesium alloy corrosion and hydrogen evolution proceed resulting in the coating damage. The following corrosion is even accelerated due to the magnesium alloy revealing to the corrosion environment. This mechanism was observed also in [47] and [48].

## 4. Conclusions

The study of the corrosion resistance of duplex Ni-P coating deposited on AZ91 magnesium alloy was performed in various environments. Based on the experimental results, the following conclusions can be drawn:

- Ni-P coatings with final thicknesses of 25, 50, 75 and 100 µm deposited on AZ91 magnesium alloy were formed by two layers—inner low-phosphorus Ni-P layer with the average P content of 5.7 wt.% and outer high-phosphorus Ni-P layer with the average P content of 11.5 wt.%.
- Low-phosphorus inner layer has the average microhardness of 620 ± 20 HV 0.025, and the outer high-phosphorus top layer has the average microhardness of 580 ± 10 HV 0.025.
- Deposited coatings significantly improved the corrosion resistance of the AZ91 magnesium alloy.
- Potentiodynamic tests in 0.1 M NaCl solution showed that the Ni-P duplex coatings deposited on AZ91 alloy improved the alloy electrochemical corrosion properties; a significant shift of E_corr_ from −1563 mV to ~−430 mV was observed. The value of i_corr_ decreased from 6.289 µA·cm^−2^ to 0.358 µA·cm^−2^. All coatings exhibited similar electrochemical characteristics, regardless of the coating thickness.
- From the results of long-term EIS measurements, the highest value of R_p_ = 250 167 Ω·cm^2^ in 0.1 M NaCl solution after 168 h for 100 µm thick coating was determined. All layers offered the same protection against the corrosion regardless of the coating thickness, except the coating with the thickness of 75 µm reaching slightly lower values of the polarization resistance.
- The immersion tests in 3.5% NaCl solution and 10% HCl solution and salt spray tests showed that with increasing thickness of the duplex Ni-P coating, the exposition time characterizing the resistance of specimens against the corrosion was increasing. In the case of the immersion test in 10% NaOH, the exposition time reached up to 1000 h for all coated samples.

## Figures and Tables

**Table 1:** Elemental composition of AZ91 magnesium alloy [wt.%].

	Al	Zn	Mn	Si	Fe	Ni	Zr	Mg
Content	8.80	0.81	0.32	0.01	<0.01	<0.01	0.01	Balance

**Table 2:** AZ91 magnesium alloy pre-treatment and coating conditions.

Process	Parameters	Conditions
Grinding	SiC paper	No. 1200
Alkaline degreasing	Soil releasing agents	pH < 12, higher temp., 20 min
Acid pickling	Acid bath	25 °C, 5 s
Low-phosphorus electroless nickel bath	NiSO_4_·6H_2_O NaH_2_PO_2_·H_2_O complexing agent activator	1.5/2 h (14/18 µm) 60 °C pH 6.8 ± 0.1
High-phosphorus electroless nickel bath	Industrial NiChem HP 1151 (Atotech)	1.5/3/5.5/8.5 h 90 °C pH 4.7 ± 0.1

**Table 3:** Corrosion potential and corrosion current density of Ni-P coatings determined from potentiodynamic curves.

Thickness of Ni-P Coating [µm]	E_corr_ [mV]	i_corr_ [µA·cm^−2^]
AZ91 alloy	−1563	6.289
25	−425	0.358
50	−434	0.371
75	−431	0.365
100	−410	0.457

**Table 4:** Values of polarization resistance of plain AZ91 magnesium alloy and deposited Ni-P coatings in 0.1 M NaCl solution.

**Samples**	**R_p_ [ Ω·cm^2^]**					
	**5 Minutes**	**1 Hour**	**2 Hours**	**4 Hours**	**8 Hours**	**12 Hours**
AZ91 alloy	1207	3167	2588	2152	9880	3685
Ni-P 25 µm	47,787	85,110	101,112	86,969	114,169	134,782
Ni-P 50 µm	36,730	70,723	80,356	88,790	118,200	131,146
Ni-P 75 µm	18,694	26,688	26,591	24,794	22,212	31,847
Ni-P 100 µm	20,136	38,920	50,203	67,388	115,983	138,065
**Samples**	**R_p_ [ Ω·cm^2^]**					
	**24 Hours**	**48 Hours**	**72 Hours**	**96 Hours**	**168 Hours**	**−**
AZ91 alloy	5,786	5,543	9,011	1,294	11,342	−
Ni-P 25 µm	164,038	181,489	190,141	233,570	63	−
Ni-P 50 µm	120,846	201,011	214,179	264,982	218,359	−
Ni-P 75 µm	42,016	55,873	57,294	59,391	72,095	−
Ni-P 100 µm	199,510	159,432	173,856	242,660	250,167	−

**Table 5:** Exposition times in selected environments [hours].

Thickness of Duplex Ni-P Coating [µm]	Environment			
	10% HCl Solution	3.5% NaCl Solution	10% NaOH Solution	Exposition Time NSS
25	2	264	1000	96
50	5	408	1000	149
75	11	552	1000	332
100	24	792	1000	430
